# Traumatic diaphragmatic rupture, a diagnostic dilemma in the presence of eventration: a case report

**DOI:** 10.4076/1757-1626-2-6615

**Published:** 2009-09-03

**Authors:** Reyaz Ahmad Lone, Mukand Lal Sharma, Mahmood Wani, Shiraz Rather, Abdul Gani Ahangar, Fouzia Rasool, Mohd Akbar Bhat, Abdul Majid Dar, Guhlam Nabi Lone, Shyam Singh, Ishtiyaq Mir, Shabir Shah, Mubashir Shah, Mohd Lateef Wani

**Affiliations:** 1 Department of Cardiothoracic and Vascular Surgery, Sheri Kashmir Institute of Medical SciencesSoura, Srinagar, Kashmir, 190011India; 2 Department of General Surgery, Shri Maharaja Hari Singh Medical CollegeKaranagr, MS, Srinagar, Kashmir, 190011India

## Abstract

Eventration of the diaphragm is the condition where the muscle is permanently elevated, but retains its continuity and attachments to the costal margins. Traumatic diaphragmatic rupture is a recognized consequence of high velocity blunt trauma to the abdomen usually a result of motor vehicle accident. Multi-slice CT and Magnetic Resonance Imaging in the pre-operative evaluation of trauma patients, diaphragmatic rupture can be still overlooked if not evaluated with the fair degree of clinical suspicion, more so if it is associated with an eventration of diaphragm - as was in our case.

## Introduction

Traumatic diaphragmatic rupture (TDR) is a recognized consequence of high velocity blunt trauma to the abdomen usually a result of motor vehicle accident (MVA). It has been reported in upto 5% of thoracoabdominal trauma patients. Early recognition of TDR is of utmost importance. Despite the development and availability of new gadgets including multi-slice CT and Magnetic Resonance Imaging in the pre-operative evaluation of trauma patients, diaphragmatic rupture can be still overlooked if not evaluated with the fair degree of clinical suspicion, more so if it is associated with an eventration of diaphragm - as was in our case.

## Case presentation

A 4-year-old, male child of Indian origin was referred to the emergency department of Sheri Kashmir Institute of Medical Sciences, the tertiary care institute of the state with a history of being in a road traffic accident causing chest and abdominal trauma. At the peripheral centre where the child was first seen resuscitation had been started and a chest tube had been placed on the right side and 200 ml of blood had been drained. The child was referred because of history of eventration of the diaphragm on the same side. At admission the child was semiconscious, pale, had features of respiratory distress with a rate of 54 breaths per minute and was in shock with no palpable peripheral pulse and an unrecordable BP. Immediately the child was intubated and further lines were set up to enable resuscitation. Secondary survey revealed bruise over the right chest, decreased air entry on the right side, central placed mediastinal structures and normal heart sounds. Rest of his examination was normal.

**Figure 1.**
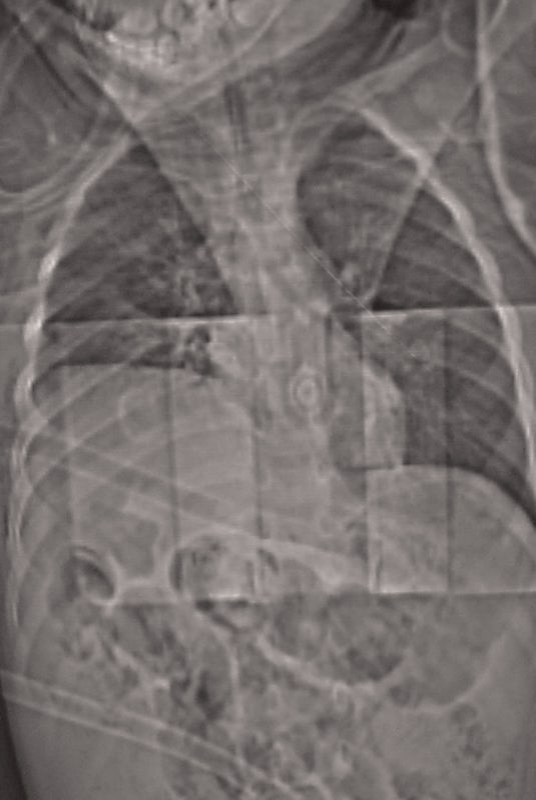
X-ray chest showing elevated right dome of diaphragm.

**Figure 2.**
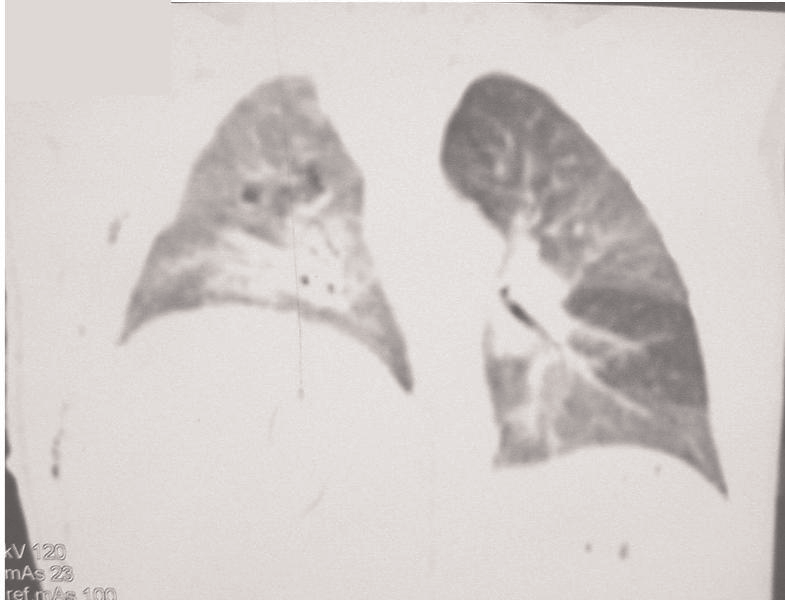
CT scan showing smooth outline elevated right hemidiaphragm.

After resuscitation the patient was taken for CT scan of abdomen and chest. CT showed free fluid in the abdominal cavity, a right sided retroperitoneal haematoma, a right lung contusion, a right haemothorax and the presence of right sided chest tube (Figure 1). The diaphragm was noted at higher level but it could not be determined whether there was any injury to it (Figure 2). Subsequently the patient was subjected to exploratory laparotomy because a TDR could not be ruled out by imaging. At surgery note was made of complete rupture of the right dome of the diaphragm with almost the whole liver in the right chest. The retroperitoneal haematoma was found to be non-expanding and about 300 ml of blood was found in the abdominal cavity. The right lower lobe of lung was contused, the pericardium was torn on the right side and its edges were bleeding. The pericardial edges were cauterized, the liver returned to the abdominal cavity and the diaphragm repaired with 2/0 prolene interrupted mattress sutures.

The patient was electively ventilated for 24 hours after which he was extubated and shifted to ward. On the second postoperative the patient started draining chyle (400-500 ml/day) from the right chest tube, confirmed by biochemical analysis. His drainage subsequently decreased by the 10^th^ day after which the chest tube was removed and the patient sent home. Presently the child is doing well and is on follow up.

## Discussion

Eventration of the diaphragm is the condition where the muscle is permanently elevated, but retains its continuity and attachments to the costal margins. It was first described by Jean Louis Petit in 1774 during a post-mortem examination [1]. Eventration is a rare, with a reported incidence of 1 in 1400 cases [2] and appears to be due to a congenital abnormality of the pleuroperitoneal membrane. Newborns with eventration may present with respiratory distress requiring mechanical ventilation, however it may remain asymptomatic and often requires no treatment. This condition may be confused with TDR in the trauma patient. Early differentiation and recognition of TDR is critical, as a delay in diagnosis is implicated in increased morbidity and mortality. A high index of suspicion, good past history and past and present imaging should aid in early and definitive diagnosis.

Diaphragmatic rupture is most commonly reported after trauma, either penetrating or blunt where the incidence is reported up to 6% [3]. Diaphragmatic rupture is left-sided in 70 to 90 percent of cases [4]. Right-sided tears are eight times less common than left-sided tears [5]. Blunt trauma secondary to a motor vehicle accident is the most common cause of closed rupture of the hemidiaphragm, here a massive force is applied to the upper abdomen or the lower chest, resulting in sudden upward compression [4]. The sudden increase in intra-abdominal pressure relative to the intrathoracic pressure results in a pressure gradient across the diaphragm. Even a violent cough has been reported to cause TDR.

Most TDR are longer than 10 cm and occur in the posterolateral aspect of the hemidiaphragm; this site is structurally weak because of its origin from the pleuroperitoneal membrane. The size of the defect as well as the size and physical characteristics of the abdominal viscera adjacent to the defect, determine whether visceral herniation will occur. While left-sided lacerations result in large herniation of abdominal contents into the left hemithorax, right-sided tears more frequently are not accompanied by herniated viscera, because of the protective effect of the liver [6].

If the diagnosis of TDR is missed, the mortality and morbidity may rise upto 50% due to visceral herniation and strangulation. Unfortunately diagnostic modalities are insufficient. Although chest radiography is abnormal in 85%, yet it is insensitive in depicting diaphragmatic rupture (46% sensitivity for left-sided ruptures and only 17% for right-sides ruptures [7]). On chest radiography signs are often masked by associated plural effusion, atelecteasis, pulmonary contusion or non-specific diaphragmatic elevation. For these nondiagnostic cases further evaluation is warranted. Diagnostic peritoneal lavage is un-reliable in detecting diaphragmatic injuries [8,9], though occasionally lavage fluid may exit through the chest tube and establish the diagnosis [9]. In blunt trauma, though CT is the investigation of choice with sensitivity and specificity of 61-71% and 87-100%, respectively in the diagnosis of diaphragmatic rupture [10]. Its main role is to rule out the other associated injuries, but it rarely allows identification of an isolated diaphragmatic rupture [11]. About half of the diaphragmatic disruptions are diagnosed for the first time at laparotomy or thoracotomy done for concomitant injuries [8,9] as was in our case. In diagnosing isolated case of right-sided diaphragmatic rupture other investigations including liver scintigraphy [12], intra-peritoneal instillation of technetium [13] and celiac arteriography have been suggested as being of value. Where other measures fail laparoscopy should be considered to rule out diaphragmatic injury [14].

We highlight the importance of a high degree of suspicion for diaphragmatic injury considering even a laparotomy when imaging is unable to convincingly rule out such an injury. Past history or documentation of eventration should not preclude the diagnosis of TDR and these patients should receive a comprehensive work up to avoid the catastrophic risks of missing a TDR.

## Conclusion

It is not uncommon for a practising surgeon to be misled by unusual history and false sense of security given by even high yield radiological gadgetry. It is only a surgeon being diaphragm conscious that an isolated diaphragmatic rupture is picked up even with the bizarre clinical presentation, from being completely asymptomatic to unexplained shock. Because of the great risk if the rupture is not corrected, a careful imaging assessment or laparoscopy should be obtained before discharge in any acutely injured patient who does not undergo surgical exploration.

## Abbreviations

CT: computed tomography
MVA: motor vehicle accident
TDR: traumatic diaphragmatic rupture
